# Monitoring of Aflatoxins B_1_, G_1_, B_2_, and G_2_ in Spices from Riyadh Markets by Liquid Chromatography Coupled to Tandem Mass Spectrometry

**DOI:** 10.1155/2024/8560378

**Published:** 2024-07-08

**Authors:** Mohamed Khabbouchi, Nour Guesmi, Abdulhameed bin Sultan, Abdulaziz Alkhuzayyim, Gharib Abdelhalim Abdo, Adel Alhotan, Walid Aljarbou

**Affiliations:** ^1^ Chemistry Section Riyadh Municipality Central Area Labs, Riyadh, Saudi Arabia; ^2^ General Directorate of Environmental Health Riyadh, Riyadh, Saudi Arabia

## Abstract

A total of 1053 samples of spices were collected from Riyadh markets during 2022. The contamination with aflatoxins (AF) B_1_ (AFB_1_), B_2_ (AFB_2_), G_1_ (AFG_1_), and G_2_ (AFG_2_) was determined via liquid chromatography coupled to tandem mass spectrometry. AF extraction from spices was performed using an acetonitrile-formic acid mixture. The results obtained show that the highest value for AFB1 (3.865 *μ*g/kg) was detected in the bay leaf sample, while the highest value for AFB_2_ (3.461 *μ*g/kg) was found in red chili powder. The values of AFG_1_ and AFG_2_ did not exceed 2.59 *μ*g/kg. The AF analysis shows that 24 out of 1053 samples (2.3%) contained one or more of these AFs. The highest percentage of contaminated samples was detected in black pepper. AFB_1_ was found in 21 samples of six types of spices, while AFG_2_ was detected in eight samples of four types of spices. Also, none of the samples exceeded the Saudi Food and Drug Authority and EU limits of 10 *μ*g/kg. The present research is not a comprehensive study; however, it provides valuable information on AFB_1_, AFB_2_, AFG_1_, and AFG_2_ levels in the Kingdom of Saudi Arabia spices.

## 1. Introduction

In recent years, much work has been carried out related to food contamination with mycotoxins, as they pose a real threat to human health and can cause significant economic losses [1]. Among these mycotoxins, aflatoxins (AF) B_1_, B_2_, G_1_, and G_2_ are considered to be of major concern and are the most common mycotoxins in foods, as these four types are naturally grown [2].

Dehydrated products, such as spices, may represent a suitable environment for the survival of mycotoxigenic fungi due to postharvest practices, improper storage, and conducive environmental conditions. Most spices in Saudi Arabia come from countries with tropical climates characterized by warm temperatures, unseasonal rainfalls, and high humidity, which are considered perfect conditions for the growth of fungi and AF contamination [3–6]. Moreover, most of these countries lack the necessary infrastructure to combat the fungal attacks on food commodities, causing AF problems [7]. Most spices are exposed to high temperatures during cooking; however, this is relatively ineffective in destroying AFs due to their heat-resistant nature [8, 9].

Among the four AFs, the growth of AFB_1_ in spices is considered the most common and the most dangerous, being the most potent carcinogen, mainly targeting the liver [10]. Due to the aforementioned facts, an annual National Food Monitoring Program (NFMP) was developed by the Saudi Food and Drug Authority (SFDA) to monitor compliance with the defined maximum limits (MLs) for AFB_1_, AFB_2_, AFG_1_, and AFG_2_ in food (total AFs = 10 *μ*g/kg for spice) and to assess food contaminant levels.

The determination of AF levels at low concentrations in complex matrices such as spices is a difficult task as they contain various pigments and secondary metabolites that can often be coextracted with AFs. Among the methods most commonly used for AF extraction are liquid-liquid extraction (LLE) and solid phase extraction (SPE) [11], which are expensive, tedious, and time-consuming [12]. The QuEChERS method (Qu: quick; E: easy; Ch: cheap; E: effective; R: rugged; and S: safe) is widely used due to its ease and suitability for AF extraction from different samples [13]. This method is based on extraction with acetonitrile, followed by liquid-liquid partition after the addition of salts (MgSO_4_ and NaCl). The use of appropriate analytical technology is an important step in addition to the extraction method itself. Liquid chromatography coupled with tandem mass spectrometry (LC-MS/MS) has key advantages, such as improved precision and accuracy, greater selectivity, high sensitivity, and speed [14, 15]. Therefore, interest in this technology has been increasing recently, especially in determining AF levels in food [16].

A Kingdom of Saudi Arabia (KSA) study by Hashem and Alamri [17] reported on the contamination of common spices with potential mycotoxin-producing fungi. This study showed that the predominant fungal genera encountered were *Aspergillus*, *Penicillium*, and *Rhizopus*, and the spices with a high propensity for contamination were ginger, fenugreek, fennel, garden thyme, red pepper, sweet cumin, and aniseed. On the other hand, sumac was the least contaminated spice.

The present study is aimed at monitoring AFB_1_, AFB_2_, AFG_1_, and AFG_2_ contamination levels in spices available in Riyadh public markets to establish a database that includes the AF levels in this region. Subsequently, the evaluation of the results and their compliance with existing Saudi regulations is determined. Finally, we consider the suitability of the commodities studied for human consumption as regards official maximum residual levels (MRLs).

## 2. Materials and Experimental Methods

### 2.1. Chemicals and Reagents

All solvents used during the experiments were HPLC-grade. The aflatoxin mixture B_1_, B_2_, G_1_, and G_2_ was acquired from Supelco (Tokyo, Japan). The standard solution, of different concentration levels (2.5–50 *μ*g/kg), was prepared in acetonitrile (VWR International, France) to construct calibration plots. Methanol and formic acid were obtained from Scharlau (≥99.0%, Barcelona, Spain). The water used was LC-MS grade and acquired from Thermo Fisher Scientific (Brussels, Belgium). Two tubes in the QuEChERS system (Chmlab Group, Barcelona, Spain) were used for extraction and cleanup. The first tube (tube 1) contained 4 g of magnesium sulphate (MgSO_4_), 1 g of sodium chloride (NaCl), and 1.5 g of citrate. The second tube (tube 2) contained 900 mg of MgSO_4_, 45 mg of graphitized carbon black (GCP), and 150 mg of primary secondary amine (PSA).

### 2.2. Sample Collection

In this project, more than 1053 samples of spices of different varieties were arbitrarily collected from retail markets, grocers, and supermarkets between January and December 2022 in Saudi Arabia. These were bay leaf (43), basil (45), India cardamom (70), caraway seeds (35), cilantro (13), cinnamon (27), cloves (20), red chili powder (133), cumin powder (44), meat kabsa spices (55), chicken kabsa spices (46), ginger (18), oregano (15), black pepper (150), saffron powder (45), seven spices (58), turmeric powder (85), white pepper (73), and rice spices (81).

Samples were placed in clean polyethylene bags, transported to the research laboratory using an insulated container, and analyzed upon arrival. All food samples were ground to pass through a 75 *μ*m diameter sieve to prepare a uniform particle size.

### 2.3. Extraction and Cleanup

Aflatoxins were extracted from spices according to the QuEChERS method with some modifications [18, 19]. Ten grams of ground and homogenized samples was mixed with 10 ml of acetonitrile-formic acid (98 : 2; *v*/*v*) in a 50 ml extraction tube (tube 1). The reactions were carried out under vigorous stirring for 7 min. The solutions were centrifuged at 4000 rpm for 5 min, depending on the type of sample. Subsequently, 6 ml of supernatant was transferred to tube 2 and shaken for 3 min [12, 20]. Then, the supernatant was centrifuged at 4000 rpm for 3 min and filtered through a nylon polyamide filter (0.45 *μ*m), and 0.5 ml of the supernatant was transferred to a 2 ml vial. Ten microliters of this sample was injected into the LC-MS.

### 2.4. LC-MS/MS Methodology

Detection was performed using an LC-MS/MS 8040 equipped with an electrospray ionization source (ESI) and a triple quadrupole mass spectrometer (Shimadzu UFLC, Kyoto, Japan). The LC-MS/MS conditions are shown in Table 1. The LC-MS 8040 was coupled to an LC−30 AD pump, a CTO−30A column oven, a DGU-20A5R degasser, a SIL−30 AC autosampler, and a CBM−20A system controller. LabSolutions software was used for control and data processing. Separation was achieved on a Shimadzu Symmetry C18 column (150 mm × 4.6 mm inner diameter; Waters, Wexford, Ireland) at 50°C. Before and after aspiration of each sample, the autosampler was rinsed with a methanol : water mix (1 : 1, *v*/*v*).

#### 2.4.1. Linearity

To evaluate the linearity of the method, five concentration levels of AF standard solutions (2.5, 5, 10, 25, and 50 *μ*g/kg) were prepared by diluting the stock standard solution with acetonitrile. Five injections from each experiment were analyzed, and the average was taken in the calculation.

#### 2.4.2. Recovery and Precision

To confirm that the analytical method was suitable for its intended purpose, its accuracy was validated by experiments involving repeatability and intermediate precision. These were carried out with noncontaminated typical samples by spiking the 5 g of samples with either 20 *μ*l, 50 *μ*l, 200 *μ*l, or 500 *μ*l of the aflatoxin standard solutions using a digital pipette. These standards were equivalent to levels of 1 *μ*g/kg, 2.5 *μ*g/kg, 10 *μ*g/kg, and 25 *μ*g/kg analytes, respectively. Five replicates were used to determine recoveries on the same day and after three days. The assay for each analysis was determined, and the % relative standard deviation (RSD) was calculated.

#### 2.4.3. Specificity

The specificity of the LC-MS method was determined by measuring the ability to separate the analyte peak from other component peaks in the sample. In this project, the specificity evaluation of AFB_1_, AFB_2_, AFG_1_, and AFG_2_ was performed by separately injecting 10 *μ*l of standard and sample solutions into the chromatographic system. The area and retention time of the standard peak were compared with those of the sample peak.

#### 2.4.4. Limit of Detection (LOD) and Limit of Quantification (LOQ)

The limit of detection (LOD) and limit of quantification (LOQ) of AFB_1_, AFB_2_, AFG_1_, and AFG_2_ were determined by analyzing different solutions of AF mixtures and measuring the signal-to-noise ratio for each analyte. The LOD is the concentration of the sample resulting in a signal-to-noise ratio of approximately 3 : 1 [21, 22]; the LOQ is the concentration resulting in a signal-to-noise ratio of about 10 : 1, with an RSD < 10% after analysis in triplicate [16, 23].

## 3. Results and Discussion

### 3.1. Validation of the Analytical Method

**Figure 1:**
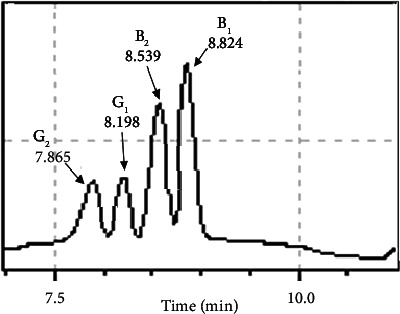
The chromatogram of aflatoxin standard solutions using LC-MS/MS.

A good linearity was achieved within the AF concentration range of 2.5–50 *μ*g/kg (Table 2). The coefficients of correlation were *R*^2^ = 0.9994, 1.000, 0.9997, and 0.9993, corresponding to AFB_1_, AFB_2_, AFG_1_, and AFG_2_, respectively (Table 2). The retention times for AFG_2_, AFG_1_, AFB_2_, and AFB_1_ were 7.865, 8.198, 8.539, and 8.824 minutes, respectively (Figure 1).

The recovery study was performed on the same day and after three days by adding 1, 2.5, 5, 10, and 25 *μ*g/kg of each AF standard to uncontaminated samples (Table 3). As shown in Table 3, the mean recovery values were between 99% and 107% for AFB_1_ and AFG_1_. Recoveries ranged from 99.89% to 105.61% for AFB_2_ and from 98.02% to 105.52% for AFG_2_, demonstrating that the recovery range was acceptable as per the rate reported by the Regulation Commission of 70–110% [24]. The precision for AFs was calculated via interday and intraday (RSD, %) tests. The AF interday precision varied from 1.1% to 6.56%, while the AF intraday precision ranged from 1.01% to 8.61%. The RSD values were acceptable as they were <20% at concentration levels within the range of 0.45–10 *μ*g/kg [25].

The LODs and LOQs of the four AFs are summarized in Table 2. LODs ranged from 0.045 to 0.206 *μ*g/kg, and LOQs ranged from 0.123 to 0.425 *μ*g/kg. The LOQs obtained in this study were slightly higher or comparable to those reported by other authors. These values were considered acceptable, like those from previous research [22, 26]. A comparison of the LODs and LOQs obtained herein with the MLs set by the SFDA for AFs in spices (10 *μ*g/kg) indicates that these LODs and LOQs were low enough to permit the implementation of the proposed strategy.

### 3.2. Selection of Product Ions

**Figure 2:**
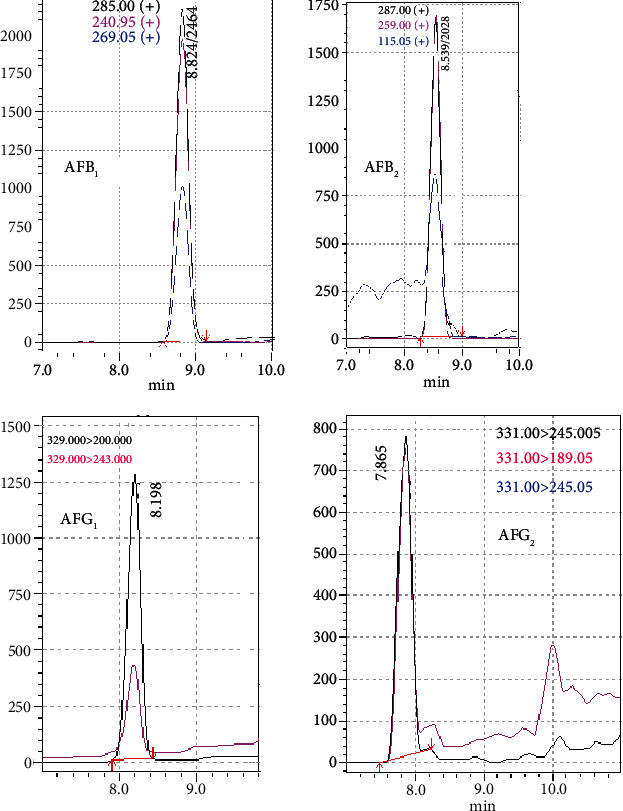
Full scan ESI production mass spectra of aflatoxin standard (AFB_1_, AFB_2_, AFG_1_, and AFG_2_).

The accuracy of AF mass measurements was assessed at various concentrations within the range of 2.5-50 *μ*g/kg to obtain mass information for the molecular ion.  Figure 2 illustrates the LC-MS mass spectrum of AFB_1_, AFB_2_, AFG_1_, and AFG_2_; the results are summarized in Table 4. From Table 4, it can be observed that there were no appreciable discrepancies in mass accuracy between the exact and observed mass (the difference being about 0.3%). Product ion selection was carried out by varying the collision energy between 10 and 40 V. Three product ions were selected for AFB_1_, AFB_2_, and AFG_2_, and two for AFG_1_, the more intense ion being the quantifier and the other the qualifier ion.

### 3.3. AF Analysis in Spice Products

In this study, 1053 samples of spices collected from Riyadh markets were surveyed for AF contamination. The results obtained are shown in Table 5. The AF levels varied in each sample; 24 out of 1053 samples contained one or more of these toxins.

The AF values (Table 5) indicated the presence of AFB_1_ in 21 samples, and AFB_2_ was present to a lesser extent, followed by AFG_1_ and AFG_2_. On the other hand, one of the bay leaf samples presented the highest contamination with AFB_1_, around 3.85 *μ*g/kg, contrasting with previous reports. Kortei et al. [27] did not detect AFB_1_ in bay leaves; Atanda et al. [28] showed that the essential oils of bay leaves reduced AF concentrations (B_1_, G_1_) of the fungus by 97.92% and 55.21%, respectively, indicating their potential AF resistance. These results indicate that our bay leaf sample may have been subjected to nonreglementary storage and/or transport conditions. To this end, the SDFA must perform continuous monitoring to detect and reduce AF contamination in various foods. The tested samples of cumin powder, ginger powder, caraway seeds, cloves, basil, saffron powder, oregano, and turmeric powder did not present any relevant fungal contamination with AFB_1_. Studies have shown that the majority of essential oils in these spices have antifungal properties [29]. This effect could also explain the low AF populations detected in seven spices powder, meat kabsa spices, and chicken kabsa spices. These spices are a mix of many crude spices, some with proven antifungal activity [29, 30].

AFG_2_ was only found in eight samples of four types of spices (Table 5), with the highest concentration (2.59 *μ*g/kg) (S14) in black pepper and the lowest (0.523 *μ*g/kg) in chicken kabsa spices (S11). AFG_1_ was detected in five types of spices: Indian cardamom, red chili powder, meat kabsa spices, chicken kabsa spices, and black pepper, in the range of 0.418-2.361 *μ*g/kg. The highest concentration was found in the Indian cardamom sample (S3). Red chili powder (S8) had the highest AFB_2_ contamination levels, 3.4601 *μ*g/kg.

No high levels of AFB_1_ and AFG_1_ were detected in red chili powder or paprika, while AFB_2_ and AFG_2_ concentrations were <3 *μ*g/kg. This result is in agreement with a previous report by Alamir et al. [31], who observed that all spice samples analyzed in KSA have an AF level of <3 *μ*g/kg. On the other hand, these results contrast with those of Aydin et al. [32]. They analyzed the B_1_ aflatoxin in 100 red pepper samples from Istanbul, finding that 18% of samples had an AFB_1_ level higher than the maximum tolerable limit (5 *μ*g/kg). Moreover, Hammami et al. [23] found that chili powder obtained from Qatari local markets contained a high level of B_1_ aflatoxin, thus confirming a previous report on AF contamination in chili powder of up to 70 *μ*g/kg [33]. Aberedew and Ayelign [34] reported on red pepper powder in an Addis Ababa market containing AFB_1_, AFB_2_, AFG_1_, and AFG_2_ levels ranging from 0.4 to 52.3 *μ*g/kg, with AFB_1_ being detected in 83% of samples with levels ranging from 1.8 to 33.3 *μ*g/kg.

**Figure 3:**
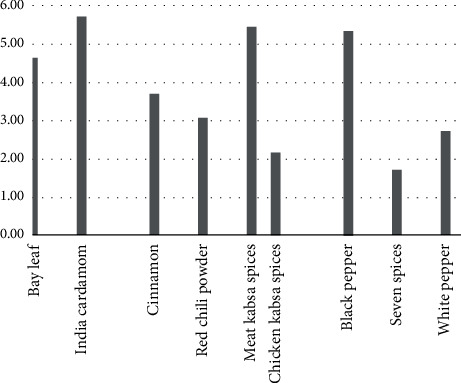
The percentage of contaminated samples in each type of spice.

The percentages of contaminated samples for each type of spice are presented in Figure 3; black pepper presented a high percentage of contaminated samples. This result is in agreement with Hammami et al. [23] and Seenappa and Kempton [35]; however, the levels detected herein are significantly lower than theirs. Black pepper is considered suitable as a substrate for AF production by *A. flavus* under artificial conditions [35]. Total AF levels in all samples were found to be below the legal limits of the SFDA (<10 *μ*g/kg) and the European Commission [36] (sum of B_1_, B_2_, G_1_, and G_2_ <10 *μ*g/kg).

## 4. Conclusions

An efficient and sensitive liquid-liquid extraction-based LC-MS/MS detection method was applied to the determination of AFB_1_, AFB_2_, AFG_1_, and AFG_2_ levels in spices. The extraction method presented an excellent mean recovery value for AFs of between 98 and 107%. The LOQs obtained, in the range of 0.123-0.425 *μ*g/kg, were lower than the maximum levels set by the EU. Linearity was demonstrated for the four AFs within the concentration ranges studied. None of the samples exceeded the SFDA limit (10 *μ*g/kg); however, some samples were contaminated with AFs. Black pepper had the highest percentage of contaminated samples. Therefore, it is important to inspect and control spices in a regular manner.

## Figures and Tables

**Table 1:** LC-MS/MS analysis conditions.

Instrument name	LC-MS 8040
Software	LabSolution
Autosampler	SIL−30 AC
Mobile phase	Water LC-MS grade-methanol 0.5% formic acid
Total flow	0.4 ml/min
Column oven temperature	50°C
Pump pressure	8.5 MPa
Injection volume	10 *μ*l
Heat block temperature	400°C
Nebulizing gas flow	1 L/min
Drying gas flow rate	20 L/min
CID gas pressure	17 KPa
(DL) temperature	250°C
Anionization mode	Binary gradient

**Table 2:** Validation data for the analysis of AFs in spice samples using LC-MS/MS.

AFs	Range (*μ*g/kg)	Linearity		LOD (*μ*g/kg)	LOQ (*μ*g/kg)
		Equation	*R* ^2^		
AFG_2_	1-50	*Y* = 5780.09 + 889.379	0.9993	0.206	0.425
AFG_1_	1-50	*Y* = 7561.67*X* − 3119.95	0.9997	0.134	0.31
AFB_2_	1-50	*Y* = 10295.7*X* + 3262.98	1.0000	0.077	0.194
AFB_1_	1-50	*Y* = 12764.5*X* + 2833.03	0.9994	0.045	0.123

**Table 3:** Mean recoveries and precision data (RSD) of aflatoxins spiked at levels in the range of 1–25 *μ*g/kg (*n* = 5).

AFs	Spiked level (*μ*g/kg)	Mean recovery (%)		Precision (RSD, %) (±SD)	
		Interday	Intraday	Interday	Intraday
AFG_2_	1	108.74	107.41	6.05 (±0.0512)	6.67 (±0.068)
	2.5	105.52	104.86	5.61 (±0.144)	5.91 (±0.151)
	5	102.8	102.24	2.49 (±0.129)	2.59 (±0.134)
	10	98.02	98.12	4.18 (±0.409)	4.25 (±0.416)
	25	99.45	100.03	1.13 (±0.28)	1.01 (±0.25)

AFG_1_	1	106.72	106.68	6.56 (±0.048)	5.61 (±0.0395)
	2.5	104.008	104.132	4.93 (±0.125)	5.2 (±0.157)
	5	102.888	102.54	2.66 (±0.133)	2.14 (±0.107)
	10	100.43	99.86	2.24 (±0.224)	2.06 (±0.206)
	25	101.76	101.22	2.16 (±0.54)	2.33 (±0.582)

AFB_2_	1	104.2	103.97	3.27 (±0.026)	3.34 (±0.034)
	2.5	105.3	105.61	4.16 (±0.106)	3.66 (±0.093)
	5	103.63	103.47	4.00 (±0.201)	4.09 (±0.205)
	10	99.89	100.202	1.18 (±0.117)	1.19 (±0.118)
	25	100.88	100.53	1.1 (±0.275)	1.51 (±0.377)

AFB_1_	1	106.96	106.74	1.78 (±0.019)	1.7 (±0.013)
	2.5	103.72	103.71	3.89 (±0.098)	3.98 (±0.1)
	5	102.178	102.69	4.23 (±0.212)	4.01 (±0.232)
	10	99.074	100.03	2.53 (±0.252)	2.16 (±0.215)
	25	100.085	99.981	2.14 (±0.535)	2.71 (±0.677)

**Table 4:** Aflatoxin database table of aflatoxins B_1_, G_1_, B_2_, and G_2_.

Molecular formula	Aflatoxin	Retention time (min)	Calculated mass (m/z)	Observed mass (m/z)	Product ions (m/z)
C_17_H_12_O_6_	B_1_	8.824	312.0634	313.000	285.00
					240.95
					269.05

C_17_H_14_O_6_	B_2_	8.539	314.0790	315.000	287.00
					259.00
					115.05

C_17_H_12_O_7_	G_1_	8.198	328.0583	329.000	200.00
					243.00

C_17_H_14_O_7_	G_2_	7.865	330.0740	331.000	245.005
					189.05
					245.05

**Table 5:** The maximum aflatoxin values (±SD) detected in each spice.

Sample name	Sample code	Sample (*n*)	AFB_1_	AFB_2_	AFG_1_	AFG_2_
Basil	S_2_	45	N.D	N.D	N.D	N.D
Bay leaf	S_1_	43	3.856 (±1.063)	2.743 (±1.188)	N.D	N.D
Black pepper	S_14_	150	1.438 (±0.638)	1.461 (±0.498)	0.438 (±0.103)	2.59 (±1.814)
Caraway seeds	S_4_	35	N.D	N.D	N.D	0.787 (±0.119)
Chicken kabsa spices	S_11_	46	N.D	0.842 (±0.522)	1.317	0.522 (±0.182)
Cilantro	S_5_	13	N.D	N.D	N.D	N.D
Cinnamon	S_6_	27	N.D	0.822 (±0.43)	N.D	N.D
Cloves	S_7_	20	ND	N.D	N.D	N.D
Cumin powder	S_9_	44	N.D	N.D	N.D	N.D
Ginger	S_12_	18	N.D	N.D	N.D	N.D
India cardamom	S_3_	70	0.742 (±0.215)	0.342 (±0.101)	2.361 (±1.015)	N.D
Meat kabsa spices	S_10_	55	1.0154 (±0.841)	N.D	1.21 (±0.262)	N.D
Oregano	S_13_	15	ND	N.D	N.D	N.D
Red chili powder	S_8_	130	1.532 (±0.963)	3.461 (±1.361)	0.938 (±0.401)	N.D
Rice spices	S_19_	81	N.D	N.D	N.D	N.D
Saffron powder	S_15_	45	N.D	N.D	N.D	N.D
Seven spices	S_16_	58	N.D	0.8	N.D	N.D
Turmeric powder	S_17_	85	N.D	N.D	N.D	N.D
White pepper	S_18_	73	0.87 (±0.313)	0.358 (±0.12)	N.D	1.06 (±0.40)
Total	19	1053				

## Data Availability

The data used to support the findings of this study are included within the article.
